# No increase in inflammation in late-life major depression screened to exclude physical illness

**DOI:** 10.1038/s41398-022-01883-4

**Published:** 2022-03-24

**Authors:** Eline T. Luning Prak, Thomas Brooks, Walid Makhoul, Joanne C. Beer, Ling Zhao, Tommaso Girelli, Carsten Skarke, Yvette I. Sheline

**Affiliations:** 1 Department of Pathology and Laboratory Medicine, Perelman School of Medicine, University of Pennsylvania, Philadelphia, PA USA; 2 Institute for Immunology, Perelman School of Medicine, University of Pennsylvania, Philadelphia, PA USA; 3 Center for Neuromodulation in Depression and Stress (CNDS), Perelman School of Medicine, University of Pennsylvania, Philadelphia, PA USA; 4 Institute for Translational Medicine and Therapeutics (ITMAT), Perelman School of Medicine, University of Pennsylvania, Philadelphia, PA USA; 5 Department of Biostatistics, Epidemiology and Informatics, University of Pennsylvania, Philadelphia, PA USA; 6 Department of Medicine, Perelman School of Medicine, University of Pennsylvania, Philadelphia, PA USA; 7 Departments of Psychiatry, Radiology, Neurology, Perelman School of Medicine, University of Pennsylvania, Philadelphia, PA USA

**Keywords:** Depression, Molecular neuroscience

## Abstract

Depression is a common and debilitating disorder in the elderly. Late-life depression (LLD) has been associated with inflammation and elevated levels of proinflammatory cytokines including interleukin (IL)-1β, tumor necrosis factor-alpha, and IL-6, but often depressed individuals have comorbid medical conditions that are associated with immune dysregulation. To determine whether depression has an association with inflammation independent of medical illness, 1120 adults were screened to identify individuals who had clinically significant depression but not medical conditions associated with systemic inflammation. In total, 66 patients with LLD screened to exclude medical conditions associated with inflammation were studied in detail along with 26 age-matched controls (HC). At baseline, circulating cytokines were low and similar in LLD and HC individuals. Furthermore, cytokines did not change significantly after treatment with either an antidepressant (escitalopram 20 mg/day) or an antidepressant plus a COX-2 inhibitor or placebo, even though depression scores improved in the non-placebo treatment arms. An analysis of cerebrospinal fluid in a subset of individuals for IL-1β using an ultrasensitive digital enzyme-linked immunosorbent assay revealed low levels in both LLD and HC at baseline. Our results indicate that depression by itself does not result in systemic or intrathecal elevations in cytokines and that celecoxib does not appear to have an adjunctive antidepressant role in older patients who do not have medical reasons for having inflammation. The negative finding for increased inflammation and the lack of a treatment effect for celecoxib in this carefully screened depressed population taken together with multiple positive results for inflammation in previous studies that did not screen out physical illness support a precision medicine approach to the treatment of depression that takes the medical causes for inflammation into account.

## Introduction

There is substantial literature reporting elevated inflammatory markers in depression (reviewed in Miller 2009; Beurel 2020) [1, 2]. Compared with nondepressed individuals, those with major depressive disorder (MDD) have elevations of inflammatory cytokines in peripheral blood and cerebrospinal fluid (CSF) [3, 4]. Increased cytokines include interleukin (IL)−1, C-reactive protein (CRP), tumor necrosis factor-alpha (TNFα), and IL-6 [5]. Patients with MDD, particularly those with late-life depression (LLD), also have increased oxidative stress [1, 6] and elevations in circulating acute phase reactants, including nitric oxide synthase and prostaglandins [6–8].

The association of inflammatory markers with LLD could point to a causal role for inflammation in the pathogenesis of LLD. In support of a causal association, IL-6 has been shown to interfere with the production of serotonin from tryptophan by increasing the breakdown of tryptophan, thus reducing serotonin levels (and increasing depression risk) and preferentially increasing the synthesis of kynurenine and its neurotoxic metabolites, 3-hydroxykynurenine and quinolinic acid. IL-6 drives this metabolic shunt and IL-10 partially counters it [1]. Proinflammatory factors including cytokines were reported to be associated with an increased risk of developing depressive symptoms [9, 10]. In the case of prolonged exposure to cytokines, there is an increased risk of becoming depressed, with ~25% of patients with chronic elevations of interferon (IFN)-α experiencing symptoms of MDD [11]. In addition, Maes and colleagues [1, 3] observed that depression is often accompanied by increased oxidative stress and lipid peroxidation.

An alternative explanation for the association of LLD and inflammation is that inflammation might result as a consequence of LLD or inflammatory conditions that “travel together” with LLD. IL-6, for example, has been called the “gerontologist’s cytokine” by William Ershler, who proposed that this cytokine regulates a key human aging pathway [12], given the consistent association between elevated plasma IL-6 and poor health outcomes. Increased plasma IL-6 is a risk factor for many diseases of aging, including hypertension, atherosclerosis, cardiovascular ischemia, and type 2 diabetes, all of which are more common in LLD [13]. Thus LLD patients with comorbid illness may represent a subset with elevated inflammation. IL-6 is also associated with sleep impairment, fatigue, and cognitive dysfunction [14]. Overall, MDD is associated with a significant reduction in lifespan, in part due to suicide and in part due to the association with comorbid major medical disorders, including cardiovascular disease and stroke, autoimmune disease, diabetes, and cancer [15–17].

Given the high rates of comorbid illness in patients with MDD, we sought to disassociate the links among depression, medical illness, and inflammation by carefully screening out patients with known inflammatory-associated illness. This approach allowed us to determine, in the absence of known comorbid illness, whether depression was associated with higher rates of inflammation. In addition to determining baseline levels of inflammation in MDD vs. controls, we sought to determine whether treatment with antidepressants had anti-inflammatory effects. Finally, we sought to determine whether antidepressants supplemented with anti-inflammatory medication, celecoxib, resulted in additional antidepressant benefits.

## Materials and methods

### Patient recruitment and study entry criteria

**Fig. 1:**
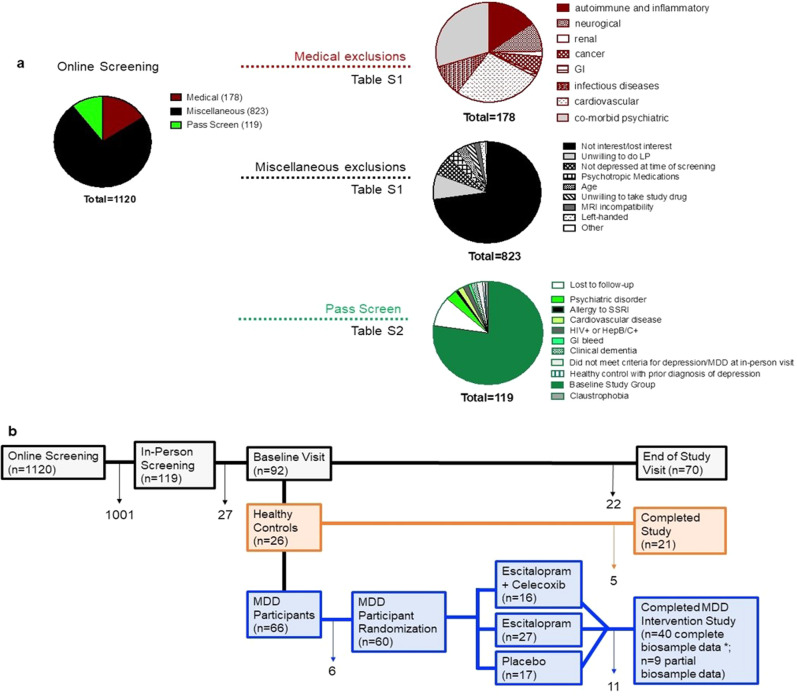
Overview of study participant groups, screening, and cohorts. **a** 1120 individuals were screened online. Of those, 178 were excluded for medical reasons (red chart and see Table S1 for further details) and 823 were excluded for miscellaneous reasons (black chart and see Table S1 for further details). The remaining 119 individuals passed the screen (green). Of those, additional individuals withdrew or were excluded upon further review (see Table S2 for details). **b** Numbers of individuals for screening, baseline, and randomization visits as well as the final subject numbers. Arrows indicate the number of individuals who withdrew or were excluded at various stages of the study. MDD = major depressive disorder. Of the *n* = 49 who completed the study *n = have complete biosample data sets. Of these 40, *n* = 18 escitalopram, *n* = 12 escitalopram + celecoxib, *n* = 10 placebo.

Our goal was to recruit a cohort of older individuals with MDD without comorbid conditions associated with inflammation. The study was approved by the University of Pennsylvania Institutional Review Board. Participants were recruited through University of Pennsylvania clinics and surrounding communities using IRB-approved flyers, radio and subway ads, and Facebook posts. A total of *n* = 1120 participants were screened remotely through a center-wide screening form, either self-report or over the phone, to identify general exclusionary factors. Medical records, where available, were screened as well to identify exclusion criteria. Miscellaneous reasons (*n* = 823) and disqualifying medical conditions (*n* = 178) determined exclusion from study participation for *n* = 1001 participants, Fig. 1a and Table S1.

Informed consent was obtained from *n* = 119 participants (*n* = 87 MDD, *n* = 32 HC) and diagnostic status was clarified through medical history and prescription drug intake review (Table S2). Psychiatric diagnoses (SCID-5), depressive symptomatology with the Montgomery Asberg Rating Scale (MADRS), Hamilton Depression Rating Scale (HDRS), and Depression History form were assessed by the study physician. The Montreal Cognitive Assessment (MoCA) (≥26/30 defined as normal) ruled out cognitive impairment. Self-report questionnaires assessed events related to stress and abuse (Perceived Stress Scale & Life Event Stress Scale, Maltreatment and Abuse Chronology of Exposure (MACE) Scale). Vital signs, EKG and biosampling for rheumatoid factor, and hepatitis screening concluded screening procedures. At the baseline visit, *n* = 92 (*n* = 63 MDD, *n* = 29 HC) were evaluated, and 70 (*n* = 49 MDD, *n* = 21 HC) completed all study visits (Fig. 1b). The randomization scheme and participants’ compliance produced allocation of *n* = 21 participants to escitalopram with *n* = 18 completing baseline and study end biomarker assessments, while for the escitalopram/celecoxib arm, these numbers amounted to *n* = 13 and *n* = 12, respectively, and for the placebo arm to *n* = 11 and *n* = 10, respectively. In cases where group sizes deviate from the above due to missing data, we report this where applicable in the results.

### Compliance

Compliance with study medication (verum & placebo) was assessed by pill count. The study coordinator dispensed and counted the study medication provided by the Penn Investigational Drug Service to the participant during scheduled study visits at the beginning of week 1, week 2, and week 4.

### Cardiovascular safety

For blood pressure and heart rate readings, all repeated values at the same visit were averaged. To compare trends in blood pressure and heart rate, differences from the baseline visit were computed. The area under the curve (AUC) is reported for each systolic blood pressure, diastolic blood pressure, and heart rate across the values in weeks 1, 2, 4, and 6. Missing values were filled by linear interpolation. A *t* test with Welch’s correction was applied to the AUC values in the ESC group (*n* = 6) versus the ESC + Celecoxib groups (*n* = 13).

### Clinical blood sample collection and processing

In all, 20 mL peripheral venous blood was collected into EDTA tubes for isolation of plasma and 4 mL of blood was collected into SST (serum separator tubes) for isolation of serum. The EDTA tubes were spun at 310 × *g* for 15 minutes, the SSTs were spun at 1000 × *g* for 15 min, and the plasma or serum was collected, aliquoted, and stored in the −80°C freezer until use.

### Immunological assays

In this study, we aimed to have 80% power to detect at least one analyte (out of 29) at 1 SD difference from controls at an FDR of <0.05. Peripheral blood cytokine levels were measured using multiplex bead arrays, high-sensitivity ELISA, or Single molecular array (Simoa) assays in the Human Immunology Core at the University of Pennsylvania. Samples were assayed using a human 29-plex cytokine/chemokine magnetic bead microsphere immunoassay panel (HCYTMAG-60K-PX29, MilliporeSigma; Burlington, VT) following the manufacturer’s instructions. Samples were run in duplicate and fluorescence data were acquired using Luminex® xPONENT® 4.2 on a FlexMAP3D Luminex instrument. IL-1β levels were also measured using an ultrasensitive digital ELISA (Simoa Cat #101605, #101622, # 101580 from Quanterix; Billereca, MS) in CSF and on selected serum/plasma samples following the manufacturer’s specifications. Samples were tested in duplicate, and data were acquired on a Simoa HD-1 analyzer [18].

### Cytokine data processing and analysis

Average cytokine or chemokine mean fluorescence intensity values (MFIs) were converted to estimated concentrations (pg/mL) through interpolation on a standard curve for each cytokine using Bio-Plex Manager software (v. 6.1, Bio-Rad, Hercules, CA). MFI values that were negative after subtraction of the blank were listed as 0. If the measurement was negative, 0, or was otherwise below the analytical sensitivity of the assay (“out of range” low, as indicated by the data analysis software), an arbitrary value of 0.001 pg/mL was used instead of 0 pg/mL for statistical purposes (to facilitate log-scale comparisons). Baseline differences in median MFI and concentration measures between MDD and HC groups were compared for each individual analyte using two-tailed Wilcoxon rank-sum tests with uncorrected *p* values < 0.05 considered statistically significant. To evaluate the aggregate measure across cytokines in an individual sample, the mean and standard deviation (SD) for each analyte were computed using the HC baseline MFI data. Fold change by SD relative to the HC group were visualized on heat maps, using similar methods to those described previously [19] Interpolated concentration, MFI data, and fold change data were visualized using Prism software v.9.1.2, GraphPad, San Diego, CA.

### Longitudinal changes in MADRS scores

MADRS scores were assessed at baseline and final visits. Subjects within each treatment group had baseline and final MADRS scores compared by a Wilcoxon signed-rank test to assess improvement in depression levels within each treatment group. The healthy controls were not examined as all MADRS scores were very low (4 or less). To assess the effect of escitalopram, all subjects receiving escitalopram (regardless of celecoxib treatment) were compared to those receiving placebo. An analysis of covariance (ANCOVA) model of the final MADRS scores by treatment (escitalopram or not) with baseline score as a covariate was performed.

## Results

### Study participants

We assembled a carefully controlled group of MDD and HC individuals. Stringent inclusion criteria compiled an MDD cohort without comorbid conditions associated with systemic inflammation (Fig. 1a, b; Table 1). MDD and HC individuals did not differ significantly with respect to the average age, race, sex, and other covariates (Table 2).

**Table 1:** Inclusion and exclusion criteria.

Inclusion criteria	Visit	Assessments
Age 50–80, right-handed male or female, any race	Screening survey	Screening survey
Absence of clinical dementia	In-person visit	Clinical Dementia Rating Scale (CDRS)
English speaking	Screening Survey	Screening survey
Blood pressure not exceeding 150/90 mmHg, treated, or untreated	In-person visit	Vitals and electrocardiogram (EKG)
Normal result on liver function test	In-person visit	Blood draw
No history of ulcer disease or GI bleeding	Both	Screening survey Medical history
Weight >110 pounds	Both	Screening survey vitals
Willing to take antidepressant medication/willing to switch antidepressant medication	Screening survey	Screening survey
DSM-IV criteria for MDD^§^	In-person visit	Structured Clinical Interview for DSM-V (SCID) Overview Montgomery Asberg Depression Rating Scale (MADRS) Hamilton Depression Rating Scale (HDRS) Depression History
Exclusion criteria		
Known history of relevant severe drug allergy or hypersensitivity	In-person visit	Medical history
Does not speak English	Screening survey	Screening survey
Cannot give informed consent	In-person visit	Informed consent process
MRI contraindications (e.g., foreign metallic implants, pacemaker, claustrophobia)	Screening Survey	Screening survey
BMI > 30	Screening Survey	Screening survey
Known primary neurological disorders, such as Parkinson’s disease, Alzheimer’s disease, traumatic brain injury, cognitive impairment, or dementia	Screening Survey	CDRS medical history
Known inflammatory disease (such as systemic lupus erythematosis, known autoimmune diseases, such as multiple sclerosis, rheumatoid arthritis, Graves’ disease, Hashimoto’s disease; Screen + for rheumatoid factor, anti-nuclear antibody, HIV, Hepatitis B or Hepatitis C)	Both	Cumulative Illness Rating Scale (CIRS) Medical History Blood Draw
Clinical Dementia Rating Scale score >0	In-person visit	CDRS
Diagnosis of a chronic psychiatric illness other than MDD	In-person visit	SCID overview
Significant handicaps (e.g., uncorrected hearing or visual impairment, mental retardation) that would interfere with testing	In-person visit	Montreal Cognitive Assessment (MoCA) The 36-Item Short Form Health Survey SF (36) Disability Scale
Bleeding diathesis	In-person visit	Medical history blood draw CIRS
Severe Medical problem, which in the opinion of the investigator would pose a safety risk to the subject	In-person visit	Medical history CIRS
Clinically significant cardiovascular disease within the last 6 months (see methods)	In-person visit	Blood draw vitals Framingham Scale EKG
Clinically significant abnormalities on EKG. Primary AV block or right bundle branch block were not necessarily exclusionary	In-person visit	EKG
Current diagnosis of cancer	Both	Screening survey Medical history
Use of an investigational medicine within the past 30 days	In-person visit	Medical history
Use of Coumadin, Warfarin within the past 2 months	In-person visit	Medical history
Current treatment with psychotropic drugs or drugs that affect the CNS such as beta-blockers, mood stabilizers, antipsychotics, steroids, or nonsteroidal anti-inflammatory medications. No subjects were included in the study unless they had been off all psychotropics for at least 3 weeks, except in the case of fluoxetine, where 5 weeks off treatment was required	In-person visit	Medical history
Current alcohol or substance abuse disorder, schizophrenia or other psychotic disorder, bipolar disorder, or current OCD	Both	SCID overview DIGS summary
History of ulcer disease, Crohn’s disease, GI bleed, or anemia	Both	Screening survey Medical history
Renal insufficiency	In-person visit	Blood draw
Any other factor that in the investigator’s judgment may affect patient safety or compliance (e.g., travel distance >100 miles from this facility)	Both	
Active suicidality or current suicidal risk as determined by the investigator^§^	In-person visit	SCID overview MADRS HDRS

^§^Indicates criteria unique to depressed participants.

**Table 2:** Demographics of study cohorts.

Variables	Healthy controls	Participants with MDD			*P* value*
	(*n* = 24)	Escitalopram + celecoxib (*n* = 14)	Escitalopram (*n* = 24)	Placebo (*n* = 15)	
Mean age (years)	65.2	57.4	62.1	61.6	0.86
SD age	10.3	6.4	9.8	8.0	
Gender (male/female)	(13/11)	(6/8)	(13/11)	(8/7)	0.95
Race (white/black)	(16/8)	(8/6)	(13/11)	(8/7)	0.38
BMI	25.3 ± 2.8	26.64 ± 3.8			0.14
*Relationship*					*0.62*
Married/long-term relationship	48%	29%	27%	21%	
Divorced/separated	30%	38%	53%	36%	
Never married	22%	33%	20%	43%	
*Education*					*0.69*
Post-graduate & professional degree	48%	21%	37%	20%	
Bachelor’s degree	23%	29%	8%	20%	
Associate degree	0%	14%	17%	47%	
Some college & high school	29%	35%	38%	13%	
*Employment*					*0.79*
Full time	43%	43%	18%	13%	
Part time	14%	29%	32%	47%	
Retired	29%	7%	18%	20%	
Unemployed	9%	7%	14%	13%	
Disability	5%	14%	18%	21%	
*Salary*					*0.95*
$50,000 and above	66%	36%	20%	21%	
$30–50,000	10%	14%	25%	7%	
$0–30,000	24%	60%	55%	72%	

*Significance of differences between MDD and HC cohorts were compared using t-tests for age and BMI and using the Freeman-Halton method for the others.

### Comparison of inflammatory peripheral blood biomarkers at baseline

**Fig. 2:**
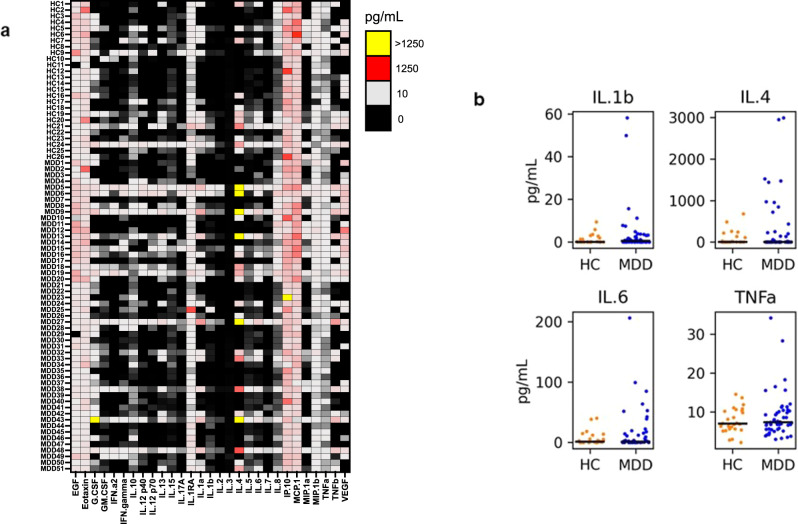
Baseline circulating cytokine profiles in individuals with major depressive disorder (MDD) vs. healthy controls (HC). **a** Heat map of cytokine profiles. Rows show the data for each individual (subject number, disease grouping), columns show each analyte. Interpolated assay values (picogram/milliliter; pg/mL) are heat mapped, with black denoting the lowest values. *EGF* epidermal growth factor, *G-CSF* granulocyte colony-stimulating factor, *GM-CSF* granulocyte-macrophage colony-stimulating factor, *IFN* interferon, *IL* interleukin, *IP-10* interferon-gamma induced protein 10 (CXCL10); *MCP1* monocyte chemoattractant protein 1, *MIP-1α* macrophage inflammatory protein 1a (CCL3), *MIP-1β* macrophage inflammatory protein 1b (CCL4), *TNF* tumor necrosis factor, *VEGF* vascular endothelial growth factor. *MDD* major depressive disorder, *HC* healthy control. **b** Comparison of selected cytokine levels in MDD (blue) vs. HC (orange). The black horizontal line denotes median values for each analyte and cohort. The sample size is *n* = 51 MDD and *n* = 26 HC. No differences were significant by a Wilcoxon rank-sum test in any of the 29 analytes (all comparisons are shown in Fig. S1, *p* > 0.1).

To determine if individuals with MDD had higher levels of systemic inflammation than HC prior to any intervention, we used a multiplex bead array assay to measure circulating levels of 29 different cytokines and chemokines at the baseline time point (see Methods and Fig. 2a shows a heat map of the data for each individual at baseline). Average analyte concentrations were computed from replicate measures and the levels of each analyte were compared between the two groups of individuals by rank-sum test (Fig. 2b shows selected cytokines, Fig. S1 shows all of the analytes, and Table S3 shows the rank-sum test results). For comparisons of all blood luminex biomarker levels at baseline between MDD and HC groups, our test (with *n* = 51 cases and 26 controls) had 86% power to detect a single analyte with a 1 SD difference from controls at an FDR < 0.05 level (with all other analytes equal between groups), correcting for the 29 analytes tested. If five of the analytes have a 0.6 SD difference from control, then power is 82% (i.e., power increases if multiple analytes differ between the groups).

No cytokines or chemokines differed significantly between MDD and HC (*p* < 0.05 by two-tailed rank-sum test), even before correction for multiple comparisons. Because there were a large number of individuals with very low levels of inflammatory markers, we also compared MDD and HC marker levels in only individuals who had non-zero levels. Here, again, none of the analytes was significantly different between the groups.

Because some individuals had mild elevations in multiple markers, we next turned to the exploratory evaluation of aggregate measures. We generated heat maps of the multiplex bead array data by MFI (Fig. S2). Using the MFI values of the HC group to compute a mean and SD, we weighted the values of each individual measurement based on its distance in SD away from the HC mean. When the binned SD distances were displayed on a heat map (Fig. S3) and scores were ranked from high to low, 17 of the top 20 aggregate scores were found in patients with MDD (Fig. S3a) but the difference in median score between MDD and HC was not statistically significant (Fig. S3b).

Because serum cytokine levels overall tended to be very low, we wondered if our failure to observe a consistent difference between individuals with and without MDD could be due to limitations in the analytical sensitivity of the multiplex bead assay. To address this issue, we evaluated IL-1β levels in MDD and controls at baseline using a digital ELISA (dELISA), an assay that is 10–100x more sensitive than the multiplex bead assay [18]. We chose IL-1β because it has previously been associated with depression [20–23] yet exhibited several values in our current assays that were out of range low. The dELISA was able to detect cytokine levels in samples that were out of range low by luminex (Fig. S4a), but no statistically significant increase in IL-1β levels was observed in MDD compared with control at baseline (Fig. S4b). For comparisons of the CSF IL-1β levels (with *n* = 5 HC and *n* = 19 MDD) power was 42% to detect a 1 SD difference between the groups. Power was determined using Monte-Carlo simulation, assuming normal distributions and performing a Wilcoxon rank-sum test.

### Improvements in MADRS scores

**Fig. 3:**
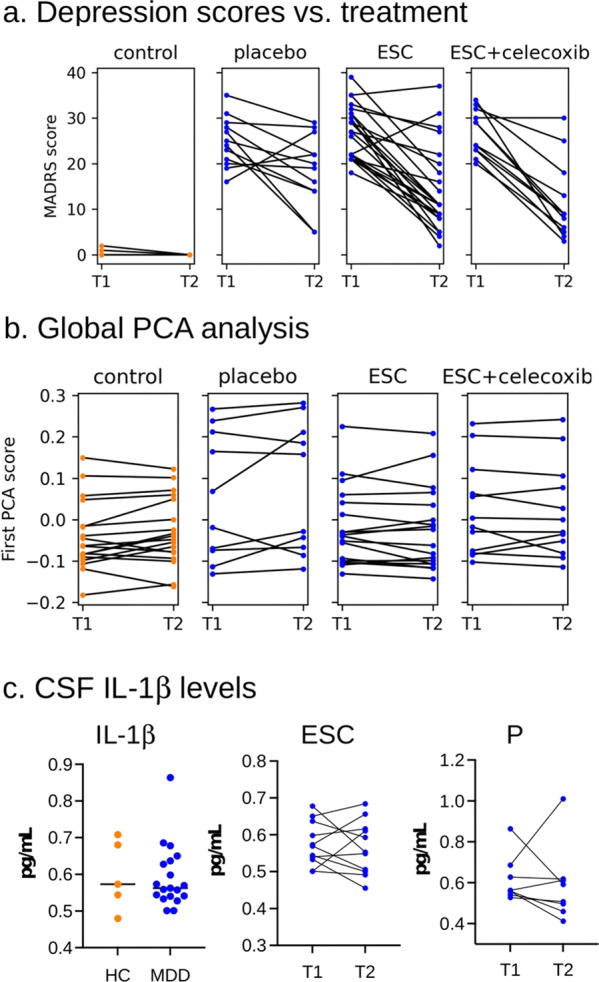
Temporal analysis of depression scores, CSF IL-1β levels, and integrated biomarkers. **a** MADRS scores at baseline and final visit. MDD patients in all groups saw significant improvement to MADRS scores (Wilcoxon signed-rank test, escitalopram *p* < 0.001 in *n* = 23, escitalopram and celecoxib *p* = 0.003 in *n* = 12, and placebo *p* = 0.034 in *n* = 12, healthy controls *n* = 23). In addition, the patients receiving escitalopram (either with or without celecoxib) saw a significant improvement over patients receiving placebo (ANCOVA *p* = 0.030, effect size = −6.34 (SE = 2.8) points, *n* = 35 with escitalopram, *n* = 12 placebo). **b** Integrated inflammatory biomarker signature at baseline compared with a final visit in the treatment and control cohorts. The scores for the top principal component were computed from inflammatory biomarker concentrations measured at T1, and then scores from T2 measurements were computed by projection according to T1 loadings. No significant changes were observed in any groups (Wilcoxon signed-rank test *p* > 0.1 for all treatments; *n* = 18 control, *n* = 10 placebo, *n* = 18 escitalopram (ESC), *n* = 12 ESC + celecoxib) and individuals’ scores were highly consistent. T1 = start of therapy; T2 = after 8 weeks of therapy. **c** IL-1β levels are low in CSF and do not change with treatment for depression. IL-1β levels in the CSF were measured by dELISA in 5 HC (orange) and 19 individuals with MDD (blue). The median level is N.S. (*p* = 0.688, Wilcoxon rank-sum test); CSF IL-1β levels do not change following treatment with ESC (20 mg/day × 8 weeks, *p* = 0.638, Wilcoxon signed-rank test); Lines interconnect samples from the same patient drawn at T1 and T2. CSF IL-1β levels do not change following treatment with placebo (P, *p* = 0.461, Wilcoxon signed-rank test); *CSF* cerebrospinal fluid, *HC* healthy control, *MDD* major depressive disorder, *ESC* escitalopram, *P* = placebo.

To verify the effectiveness of the treatments, MADRS scores were assessed at baseline and at the final visit (Fig. 3a, Table S4). MDD and HC participants who completed the study visits did not differ significantly with respect to their underlying medical conditions (Table S5). Among the MDD participants who were stratified to different treatment groups, scores were comparable between escitalopram (27.1 ± 5.6 mean ± SD), escitalopram/celecoxib (26.2 ± 4.8) and placebo (25.5 ± 5.4). MDD patients in all groups experienced significant improvement in their MADRS scores (Wilcoxon signed-rank test, escitalopram, 14.0 ± 9.1, *p* < 0.001, *n* = 23; escitalopram/celecoxib, 13.2 ± 9.9, *p* = 0.003, *n* = 12: placebo, 19.1 ± 7.9, *p* = 0.034, *n* = 12). In addition, the patients receiving escitalopram (either with or without celecoxib) saw a significant improvement over patients receiving placebo (ANCOVA *p* = 0.030, effect size = −6.34 (SE = 2.8) points, *n* = 35 on escitalopram, *n* = 12 on placebo). The lower mean MADRS score in escitalopram/celecoxib compared with escitalopram did not attain statistical significance (ANCOVA *p* = 0.42, celecoxib effect size = −2.46 (SE = 3.0) points, *n* = 12 escitalopram+celcecoxib, *n* = 23 escitalopram).

### Longitudinal changes in inflammatory peripheral blood biomarkers

Baseline and final visits of cytokine and chemokine levels were compared and were remarkably similar over time in most individuals (Fig. S5). No analyte showed significant changes from baseline after adjustment for multiple comparisons (Table S6, *p* > 0.05, Wilcoxon signed-rank test). To determine whether there were changes in aggregate inflammation levels, the top PCA component was computed in both baseline and final visit, using loadings determined by baseline alone. No treatment group had a significant change in PCA score (Wilcoxon signed-rank test, *p* > 0.1 for all treatments), and individuals’ scores were highly consistent across the two time points (Fig. 3b).

### CSF levels of IL-1β were low and did not change following treatment with escitalopram or placebo

To determine if there were elevations in intrathecal cytokines in our carefully screened cohort of MDD patients, CSF was obtained and analyzed using the dELISA assay for IL-1β from 19 cases and 5 controls. Levels of IL-1β were uniformly low in the CSF and did not differ significantly between MDD and controls at baseline (Fig. 3c, *p* = 0.688, Wilcoxon rank-sum test). Furthermore, no significant change in IL-1β levels was observed in MDD individuals after treatment with escitalopram (Fig. 3c, *p* = 0.638, Wilcoxon signed-rank test). Treatment with a placebo also did not significantly alter CSF IL-1β levels (Fig. 3c, *p* = 0.461, Wilcoxon signed-rank test).

### Cardiovascular safety

Because celecoxib has been associated with cardiovascular toxicity, we evaluated blood pressure and heart rate responses in individuals on escitalopram + celecoxib as a safety signal. Averaged blood pressure responses in individuals on escitalopram/celecoxib were higher compared to escitalopram only, but this trend was not statistically significant (*p* = 0.23 for SBP, *p* = 0.32 for DBP). The heart rate response was lower in individuals on escitalopram/celecoxib (*p* = 0.002) (Fig. S6). However, the degree of variability between screen and baseline measurements was comparable in magnitude to the effect size following treatment. Taken together, these data suggest that there is no significant cardiovascular signal using these read-outs.

## Discussion

In this study, we observed very low levels of circulating cytokines and inflammatory mediators in individuals with MDD and in controls, when study participants in both groups were carefully screened to exclude medical causes of inflammation. At baseline, MDD patients and controls both exhibited very low levels of 29 different circulating cytokines and chemokines. In many cases the levels of cytokines fell below the analytical sensitivity of the luminex assays, a finding that has also been reported in the literature when individuals with low levels of inflammation are included among the comparison groups (for example, see: [24–26]) The lack of difference between MDD and control groups was not due to poor sensitivity of the assays as high-sensitivity analysis of IL-1β levels in serum using a digital ELISA also did not reveal any difference between MDD patients and controls.

We also did not observe a significant difference in inflammatory markers between patients with MDD and controls. When MDD patients were treated with an antidepressant (escitalopram), their depression scores improved compared to placebo. However, when MDD patients were treated with escitalopram and an anti-inflammatory (celecoxib), depression scores and cytokine levels following treatment were not lower than in MDD patients who were treated with escitalopram alone. Finally, to get “closer to the source” of the inflammation, IL-1β levels were also measured in the CSF for a subset of MDD patients treated with escitalopram or placebo. Intrathecal CSF IL-1β levels were low and remained low irrespective of treatment. Taken together, these results indicate that depression in and of itself does not cause inflammation in this carefully selected patient population and that anti-inflammatory treatment of patients with MDD who do not have inflammation has no additional antidepressant benefit.

Our results contrast with many previous studies of MDD in which cytokine elevations have been reported. In multiple meta-analyses [20, 27–29] increased proinflammatory cytokines and acute-phase proteins were reported in MDD patients, with a fairly unanimous consensus of increases in IL-6, TNFα, and CRP in the blood of MDD patients compared to healthy controls [1, 30, 31]. Further, with advances in the measurement of cytokines by multiplexing [32], many additional cytokines are now evaluated [33, 34]. A recent meta-analysis of 82 studies including 3212 MDD patients and 2798 healthy controls revealed increased levels of IL-6, TNF, IL- 10, sIL-2, C-C motif chemokine ligand 2 (CCL)2, IL-13, IL-18, IL-12, IL-1RA, and soluble TNF receptor (sTNFR) in MDD patients [29]. However, patients in those studies were not excluded on the basis of physical illness associated with inflammation.

In this study, all participants were screened to exclude illnesses with known associations [35, 36] with inflammation, including inflammatory bowel disease, rheumatoid arthritis, among an extensive list. The participants included in the study, including those who completed the final study visit had medical illnesses requiring ongoing medical treatment, but these illnesses did not differ in prevalence between the HC and MDD groups. The large number of patients excluded is notable in that it points to the high rates of physical illness in most patients with depression. It has been estimated that more than half of MDD patients have associated comorbidities [37, 38] including hypertension, metabolic disorders, and chronic lower respiratory diseases [39]. Also consistent with the hypothesis that inflammatory conditions are associated with depression, a meta-analysis across 22,000 patients reported in 40 studies found a pooled prevalence of mental disorders of 36.6% in patients with chronic physical diseases [40]. Finally, an elegant study demonstrated a marked risk of becoming depressed following cytokine exposure, with ~25% of initially euthymic patients exposed to IFN-α experiencing symptoms of MDD [11].

With respect to therapy, there was no significant effect of escitalopram on inflammation levels in the current study. Further, unlike some previous studies, the addition of celecoxib to escitalopram did not change the outcome of depression response. A number of studies have supported fairly large effect sizes [41] for trials of nonsteroidal anti-inflammatory drugs and cyclooxygenase-2 (COX-2) inhibitors as antidepressants, especially as antidepressant augmenting agents, although other studies have found no antidepressant benefit for these agents [42, 43]. Further, some studies have shown that the effect of anti-inflammatory drugs on depression is dependent on the patient’s baseline inflammatory levels. This was first suggested by Raison and collaborators [44], replicated by Nettis and collaborators [45], and reviewed by Branchi and collaborators [46]. Given the low baseline levels of inflammation in our MDD cohort, it is perhaps not surprising that there was no benefit of augmenting antidepressant treatment with celecoxib.

A limitation of our study is that levels of cytokines were not measured in the medically excluded patients with inflammation to demonstrate elevations, which would have provided a more direct test of the hypothesis that physical illness rather than depression was associated with inflammation in our patient population. However, as discussed, previous studies provide ample demonstration of cytokine elevations in patients with identified inflammatory conditions. Another challenge was that patients dropped out of the study. The dropout rate in our study (24%) is consistent with dropout rates for depression studies reported in the literature (10–32%) [47–49]. Further, those who dropped out did not differ demographically from those who remained in the study and we have no evidence to suggest that individuals who dropped out differed with respect to their inflammatory profiles, because all of the individuals at baseline had low levels of inflammatory markers.

Our results add to others who argue that depression should be considered a heterogeneous disorder [46]. Our study identifies depressed individuals who do not have evidence of systemic inflammation, while other studies have focused on depressed patients with inflammation. In support of this concept of heterogeneity in MDD, a recent study [50] found distinct immunologic profiles for inflamed and non-inflamed depression, with the inflamed group characterized by increased levels of circulating neutrophils, monocytes, CD4+ T cells along with elevated IL-6 and CRP. In addition, when data-driven techniques were used to agnostically define different immunologic variants of MDD, four distinct subgroups were identified: two with increased IL-6 and CRP and more severe depression, one with predominant increases in neutrophils, and monocytes, and one with increased lymphoid cells. Together with these studies, our study supports mechanistically distinct groups of inflamed depression and an immunological distinction between inflamed and non-inflamed depression. Our findings in MDD patients with non-inflamed depression make the important point that inflammation is not an intrinsic component of depression and prompt further consideration of personalized therapeutic approaches for MDD in which both inflammation and depression are addressed.

## Supplementary information

Supplemental Material

Figure S1

Figure S2

Figure S3

Figure S4

Figure S5

Figure S6
