# Supplementary material for: No increase in inflammation in late-life major depression screened to exclude physical illness
Source: Transl Psychiatry. 2022 Mar 24;12:118. doi: 10.1038/s41398-022-01883-4 (PMC8948274; doi:10.1038/s41398-022-01883-4)
Supplement: Supplementary file 1 — Supplemental Material [file 41398_2022_1883_MOESM1_ESM.docx]

## **Supplementary Figures (Please see PowerPoint attachment)**

**Figure S1. Comparative baseline levels of individual inflammatory biomarkers in patients with MDD and HC.**

Baseline concentration values of all 29 analytes quantified in patients with MDD (blue) compared to HC (orange). No differences were significant by a rank-sum test (*p* > 0.1). Black horizontal line denotes median values for each analyte and cohort. The sample size is n=51 MDD (major depressive disorder) and n=26 HC (healthy controls).

**Figure S2: Baseline Circulating Cytokine Profiles in Individuals with Major Depressive Disorder (MDD) vs. Healthy Controls (HC).** Each row shows the data for an individual (subject number, disease grouping). Each column indicates an analyte. Assay mean fluorescence intensity (MFI) values are heat mapped, with black denoting the lowest values. Values above 5,000 are in yellow. Cytokine abbreviations are as described in **Fig. 2**.

**Figure S3: Thresholding of Baseline MFI values in Individuals with Major Depressive Disorder (MDD) vs. Healthy Controls (HC).** **a.** Each row shows the data for an individual (subject number, disease grouping). Each column indicates an analyte. Values are heat mapped based upon the mean + SD MFI values of the HC group (Baseline samples only). From these MFI data, thresholds were binned as follows: 0 (clear) < m + 1s; 1 (yellow) = m + 1s to < m + 2s; 2 (green) = m + 2s to < m + 3s; 3 (blue) = m + 3s to < m + 4s; 4 (orange) = m + 4s to < m + 5s; 5 (red) > m + 5s. **b.** Comparison of baseline aggregate MFI scores in MDD vs. HC. N.S. = not statistically significant, Wilcoxon rank-sum test on median scores. Cytokine/chemokine abbreviations are as detailed in **Fig. 2**.

**Figure S4. Circulating IL-β Analysis with a Higher Sensitivity dELISA. a.** Correlation of Luminex (main assay method) and digital ELISA (dELISA) on 5 healthy controls (HC) and 19 individuals with major depressive disorder (MDD). 12 values (open circles) are out of range (OOR) low for the luminex assay method and are assigned an arbitrary concentration of 0.001 pg/mL. All of these OOR samples had non-zero values using the dELISA. The Spearman correlation coefficient between the two methods is 0.5599 (p=0.0044). **b.** Comparison of IL-1β levels quantified by dELISA in HC vs. MDD. Differences in median values (horizontal lines) are not statistically significant (ns, p=0.07 by Wilcoxon rank-sum test, though there are only 5 individuals in the HC group).

**Figure S5. Longitudinal changes in inflammatory biomarkers on a log-scale.** All 29 analytes are shown with changes from baseline to final concentration levels in n=58 individuals in whom paired cytokine data were generated, by treatment group (n=18 controls, n=10 placebo, n=12 escitalopram (ESC) and n= 12 ESC + celecoxib). Values are shown in log-scale and values below the detection limit are omitted.

**Figure S6. Cardiovascular Safety Assessments.** Systolic (top) and diastolic (center) blood pressure and heart rate (bottom) were repeatedly assessed at clinic visits for individuals on escitalopram/celecoxib and escitalopram. Absolute blood pressure and heart rate traces (left) are displayed along baseline corrected changes over time (right). For the placebo condition, cardiovascular assessments were only available for the screen visit (left).

## **Supplementary Tables**

### **Table S1: Reasons for subject exclusion (online screening)**

| **Medical reasons** | **# of participants** |
| --- | --- |
| **Autoimmune and Inflammatory Diseases** | **27** |
| Ulcerative Colitis | 3 |
| SLE (Systemic Lupus Erythematosus) | 2 |
| Psoriasis | 1 |
| RA (Rheumatoid Arthritis) | 4 |
| Connective Tissue Disease | 1 |
| Hashimoto’s Thyroiditis | 1 |
| Polymyalgia Rheumatica | 1 |
| Grave’s disease | 1 |
| Granulomatosis and Polyangiitis | 1 |
| Multiple Sclerosis | 1 |
| Chronic Inflammatory Condition on Methotrexate | 1 |
| Chronic Inflammatory Condition on Prednisolone | 2 |
| Chronic Inflammatory Disease on NSAIDs (Non-steroidal anti-inflammatory drugs) | 7 |
| Gout | 1 |
| **Neurological Disorders** | **17** |
| Tourette’s Syndrome | 1 |
| Parkinson’s disease | 1 |
| Fahr’s Syndrome (idiopathic basal ganglia calcification) | 1 |
| Post-Concussive Brain Syndrome with memory loss, Traumatic Brain Injury | 2 |
| Intellectually Disabled / Cognitive Impairment | 2 |
| Donepezil for Alzheimer’s disease | 1 |
| Receiving medications for seizures (Anticonvulsants (Gabapentin, Pregabalin, Topiramate) | 5 |
| Sleep Apnea that requires the use of CPAP | 4 |
| **Severe Renal Disease** | **3** |
| Chronic kidney disease with glomerular filtration rate (eGFR <30) | 1 |
| Kidney Failure on Dialysis | 1 |
| Severe Kidney Disease | 1 |
| **Cancer Diagnosis** | **10** |
| Barrett’s Esophagus with dysplasia | 1 |
| Colon Cancer | 1 |
| Thyroid Cancer | 1 |
| Undergoing Cancer treatment | 6 |
| Kidney Cancer | 1 |
| **GI Problems** | **2** |
| Rectal Bleeding | 1 |
| History of intestinal bleeding, Gastric surgery | 1 |
| **Cardiovascular Diseases** | **48** |
| Congestive Heart Failure (CHF) | 1 |
| Uncontrolled High Blood Pressure | 2 |
| SVT & Cardiac Syncope | 1 |
| High Blood Pressure | 1 |
| Cardiovascular Disease & Stroke | 1 |
| Symptomatic Mitral Valve Prolapse (MVP) | 1 |
| Beta Blockers (Lopressor) | 17 |
| Blood Thinner (Warfarin, Coumadin) | 24 |
| **Infectious Diseases** | **18** |
| HIV (human immunodeficiency virus) | 14 |
| Hepatitis B/C | 3 |
| Chronic antibiotic use | 1 |
| **Comorbid Psychiatric Disorder** | **53** |
| **Bipolar Disorder** | 24 |
| **Schizophrenia Disorder/ Schizoaffective Disorder** | 12 |
| **Manic Depression** | 1 |
| **Delusional Thinking** | 1 |
| **Suicide Risk** | 1 |
| **Alcohol Abuse** | 2 |
| **Marijuana** | 8 |
| **Opiates (Methadone – Oxycodone – Hydrocodone)** | 4 |
| **Miscellaneous** | **823** |
| Unwillingness to do lumbar puncture (LP) | 66 |
| On psychotropic medications | 28 |
| Unwillingness to take the study drug | 21 |
| Not depressed at the time of screening | 52 |
| Left-handed | 14 |
| Not interested/ Lost interest (compensation,  time, distance, lost to follow-up, withdrew) | 599 |
| Age < 50 | 24 |
| MRI incompatibility (Claustrophobic or Unable to get MRI due to obesity) | 15 |
| Other various factors | 4 |

### **Table S2: Reasons for enrolled subject exclusion**

| **Reasons for Exclusion** | **Number of Participants (n=49)** |
| --- | --- |
| **From Screening to Baseline** | **27** |
| Lost to follow up | 11 |
| Mania, Hypomanic episode, Schizoid-affective disorder, Dysthymic disorder | 4 |
| Allergy to SSRI (Prozac) | 1 |
| Cardiovascular disease | 2 |
| HIV+ - Hepatitis B/C+ | 2 |
| GI bleed | 1 |
| Clinical dementia | 2 |
| Did not meet criteria for depression/MDD at the in-person visit | 2 |
| Healthy control with previous diagnosis of depression | 1 |
| Claustrophobia | 1 |
| **From Baseline to Final Visit** | **22** |
| COVID-related University Shutdown | 5 |
| Adverse events: Escitalopram Side Effects (Diarrhea, Light-headed-nausea) & severe sciatic nerve pain after the lumbar puncture | 4 |
| Unforeseen exclusionary medical reasons: Renal insufficiency-Inflammatory diseases - Neurofibromatosis | 7 |
| Unexpected Claustrophobia | 1 |
| Participant’s decision to withdraw (time commitment- relocate) | 5 |

### **Table S3: Circulating inflammatory marker comparison at baseline**

| Analyte | *p*-value | Analyte | *p*-value |
| --- | --- | --- | --- |
| EGF | 0.29 | **IL-2** | 0.72 |
| Eotaxin | 0.19 | **IL-3** | 0.62 |
| G-CSF | 0.25 | **IL-4** | 0.52 |
| GM-CSF | 0.42 | **IL-5** | 0.27 |
| IFNa2 | 0.28 | **IL-6** | 0.69 |
| IFNgamma | 0.40 | **IL-7** | 0.44 |
| IL-10 | 0.69 | **IL-8** | 0.15 |
| IL12p40 | 0.84 | **IP10** | 0.28 |
| IL12p70 | 0.28 | **MCP1** | 0.34 |
| IL-13 | 0.18 | **MIP1a** | 0.85 |
| IL-15 | 0.20 | **MIP1b** | 0.87 |
| IL-17A | 0.87 | **TNFa** | 0.69 |
| IL-1RA | 0.55 | **TNFb** | 0.68 |
| IL-1a | 0.82 | **VEGF** | 0.58 |
| IL-1b | 0.13 |  |  |

For each of the 29 analytes, the values among HC and MDD groups were compared by a Mann Whitney U rank sum test. No tests were significant even before correcting for multiple testing. N=51 MDD, 26 HC.

### **Table S4: MADRS scores by visit and group**

|  | | | |
| --- | --- | --- | --- |
|  |  |  |  |
| **baseline** |  |  |  |
| group | mean | sd |  |
| control | 1.27 | 1.64 |  |
| ESC | 27.08 | 5.56 |  |
| ESC+celecoxib | 26.21 | 4.79 |  |
| placebo | 25.53 | 5.41 |  |
|  |  |  |  |
| **Week 1** |  |  |  |
| group | mean | sd |  |
| ESC | 20.78 | 8.55 |  |
| ESC+celecoxib | 21.00 | 9.70 |  |
| placebo | 24.00 | 6.46 |  |
|  |  |  |  |
| **Week 2** |  |  |  |
| group | mean | sd |  |
| ESC | 18.95 | 11.34 |  |
| ESC+celecoxib | 19.14 | 9.28 |  |
| placebo | 23.00 | 7.41 |  |
|  |  |  |  |
| **Week 4** |  |  |  |
| group | mean | sd |  |
| ESC | 18.71 | 9.95 |  |
| ESC+celecoxib | 14.86 | 8.56 |  |
| placebo | 22.53 | 9.06 |  |
|  |  |  |  |
|  |  |  |  |
| **Week 6 (Final Visit)** | | | |
| group | mean | sd |  |
| ESC | 14.04 | 9.13 |  |
| ESC+celecoxib | 12.93 | 9.59 |  |
| placebo | 19.14 | 7.92 |  |

**Table S5: Underlying medical conditions in MDD versus HC participants who completed the study**

|  | **MDD (n out of 49), (percent)** | **HC (n out of 21), (percent)** | **P Value** |
| --- | --- | --- | --- |
| **With medical illness** | **32 (65%)** | **12 (57%)** | **0.59** |
| Coronary artery disease/atrial fibrillation | 1 (2%) | 1 (5%) | 0.51 |
| Hypertension* | 18 (37%) | 11 (52%) | 0.29 |
| Hypercholesterolemia | 11 (22%) | 4 (19%) | 1.0 |
| Hypothyroidism | 2 (4%) | 2 (10%) | 0.58 |
| Type 2 diabetes | 6 (12%) | 3 (14%) | 1.0 |
| [Gastroesophageal reflux disease (GERD)](https://www.mayoclinic.org/diseases-conditions/gerd/symptoms-causes/syc-20361940) | 5 (10%) | 0 (0%) | 0.31 |
| Pulmonary disease | 7 (14%) | 0 (0%) | 0.094 |

*Hypertension was exclusionary. However, some patients had a diagnosis of “hypertension” but on screening had BP < 150/90 and thus were included.

### **Table S6: Longitudinal inflammation biomarker p-values**

| Analyte | All | Treated | Treated, nonzero |
| --- | --- | --- | --- |
| EGF | 0.16 (58) | 0.04 (30) | 0.04 (30) |
| Eotaxin | 0.14 (58) | 0.15 (30) | 0.15 (30) |
| G-CSF | 0.57 (58) | 0.16 (30) | 0.17 (24) |
| GM-CSF | 0.6 (58) | 0.66 (30) | 0.66 (20) |
| IFNa2 | 0.5 (58) | 0.75 (30) | 0.84 (6) |
| IFNgamma | 0.25 (58) | 0.76 (30) | 0.77 (18) |
| IL-10 | 0.38 (58) | 0.95 (30) | 0.95 (29) |
| IL-12p40 | 0.14 (58) | 0.25 (30) | 0.28 (11) |
| IL-12p70 | 0.19 (58) | 0.59 (30) | 0.65 (9) |
| IL-13 | 0.31 (58) | 0.65 (30) | 0.67 (20) |
| IL-15 | 0.28 (58) | 0.83 (30) | 0.83 (30) |
| IL-17A | 0.06 (58) | 0.24 (30) | 0.28 (10) |
| IL-1RA | 0.32 (58) | 0.77 (30) | 0.77 (30) |
| IL-1a | 0.52 (58) | 0.55 (30) | 0.58 (14) |
| IL-1b | 0.33 (58) | 0.24 (30) | 0.25 (24) |
| IL-2 | 0.1 (58) | 0.35 (30) | 0.44 (5) |
| IL-3 | 0.82 (58) | 0.33 (30) | 0.33 (27) |
| IL-4 | 0.57 (58) | 0.51 (30) | 0.54 (13) |
| IL-5 | 0.99 (58) | 0.41 (30) | 0.41 (28) |
| IL-6 | 0.19 (58) | 0.53 (30) | 0.53 (28) |
| IL-7 | 0.8 (58) | 0.33 (30) | 0.37 (11) |
| IL-8 | 0.6 (58) | 0.09 (30) | 0.09 (30) |
| IP10 | 0.24 (58) | 0.35 (30) | 0.35 (30) |
| MCP1 | 0.69 (58) | 0.91 (30) | 0.91 (30) |
| MIP1a | 0.55 (58) | 0.36 (30) | 0.38 (17) |
| MIP1b | 0.25 (58) | 0.09 (30) | 0.09 (30) |
| TNFa | 0.72 (58) | 0.42 (30) | 0.42 (30) |
| TNFb | 0.62 (58) | 0.97 (30) | 1 (14) |
| VEGF | 0.89 (58) | 0.94 (30) | 0.94 (20) |

Inflammation biomarker concentration values were tested for differences between baseline and final visit among different groups by a Wilcoxon signed-rank test. The groups tested were all patients, then all patients receiving treatment (escitalopram with or without celecoxib), and those receiving treatment after dropping values below detection limits (out of range low). While EGF in the treatment groups has a nominal p-value with p<0.05, no analyte has significant changes after accounting for the multiple tests performed (adjusted Holm-Sidak p=0.72 for EGF).
