# Supplementary figures and images for: No increase in inflammation in late-life major depression screened to exclude physical illness
Source: Transl Psychiatry. 2022 Mar 24;12:118. doi: 10.1038/s41398-022-01883-4 (PMC8948274; doi:10.1038/s41398-022-01883-4)

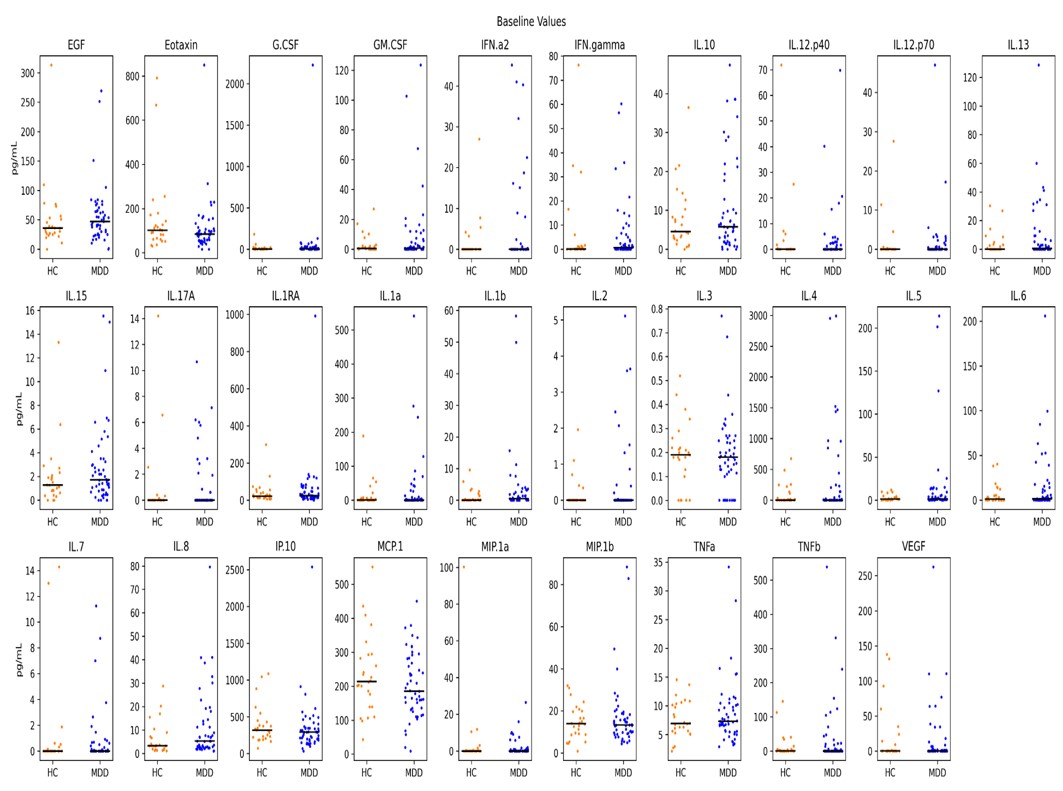

Supplement: Supplementary file 2 — Figure S1 [file 41398_2022_1883_MOESM2_ESM.jpg]

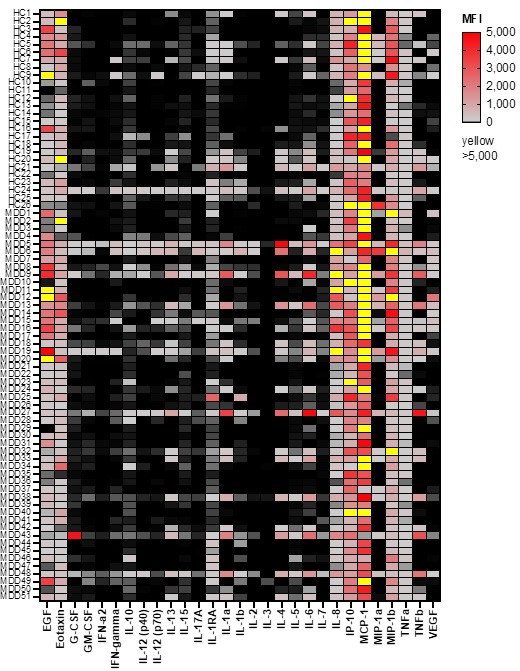

Supplement: Supplementary file 3 — Figure S2 [file 41398_2022_1883_MOESM3_ESM.jpg]

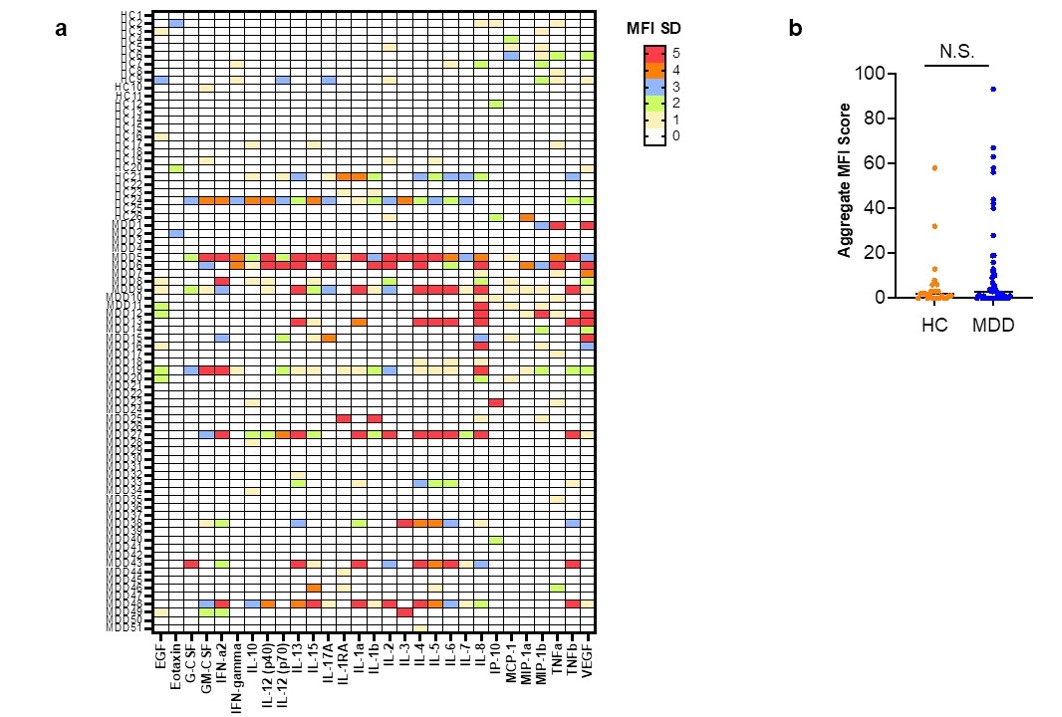

Supplement: Supplementary file 4 — Figure S3 [file 41398_2022_1883_MOESM4_ESM.jpg]

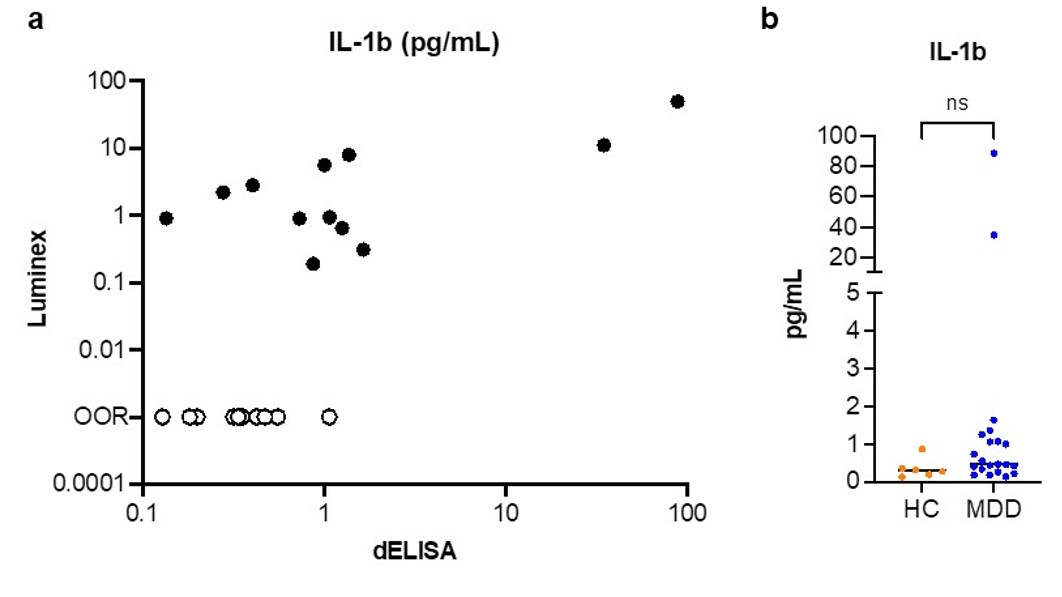

Supplement: Supplementary file 5 — Figure S4 [file 41398_2022_1883_MOESM5_ESM.jpg]

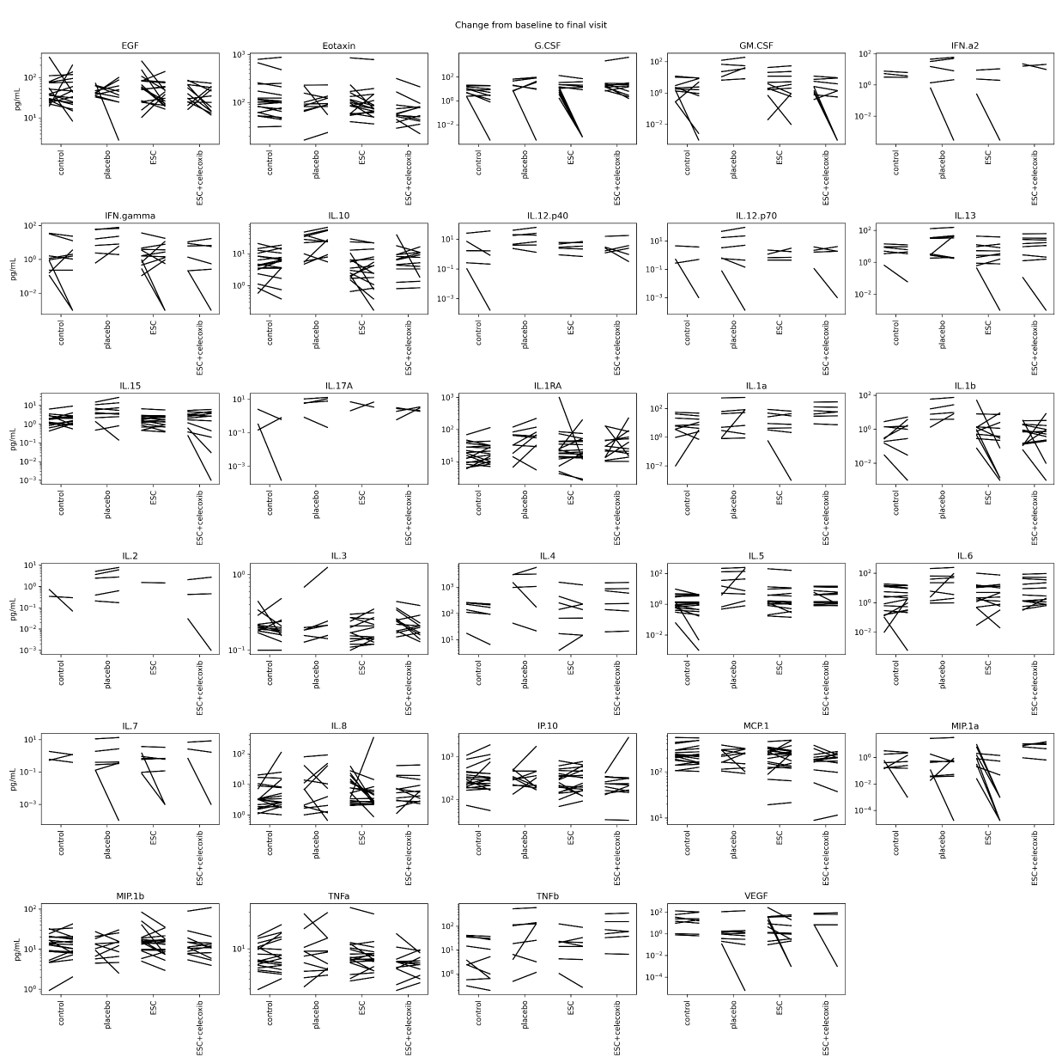

Supplement: Supplementary file 6 — Figure S5 [file 41398_2022_1883_MOESM6_ESM.jpg]

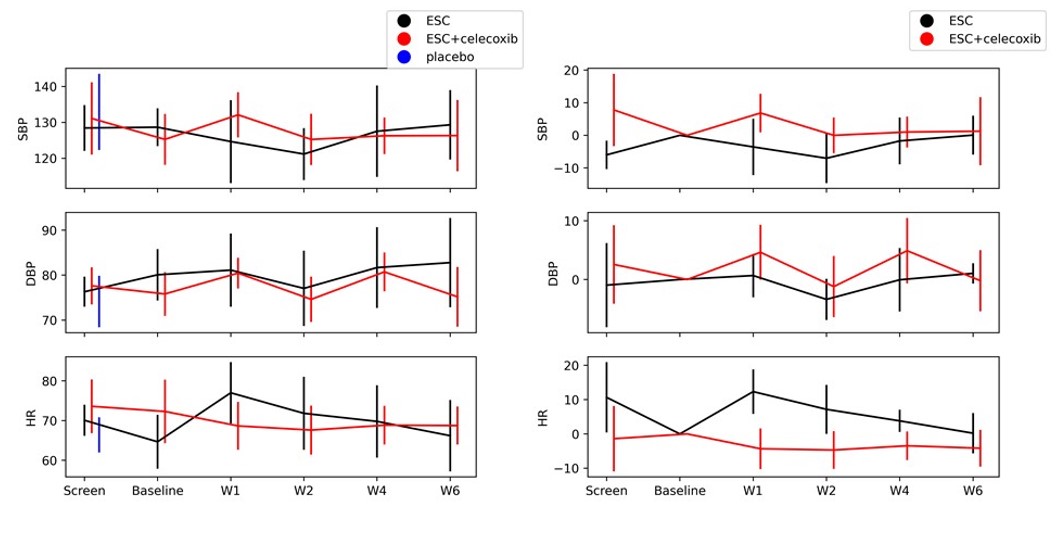

Supplement: Supplementary file 7 — Figure S6 [file 41398_2022_1883_MOESM7_ESM.jpg]
